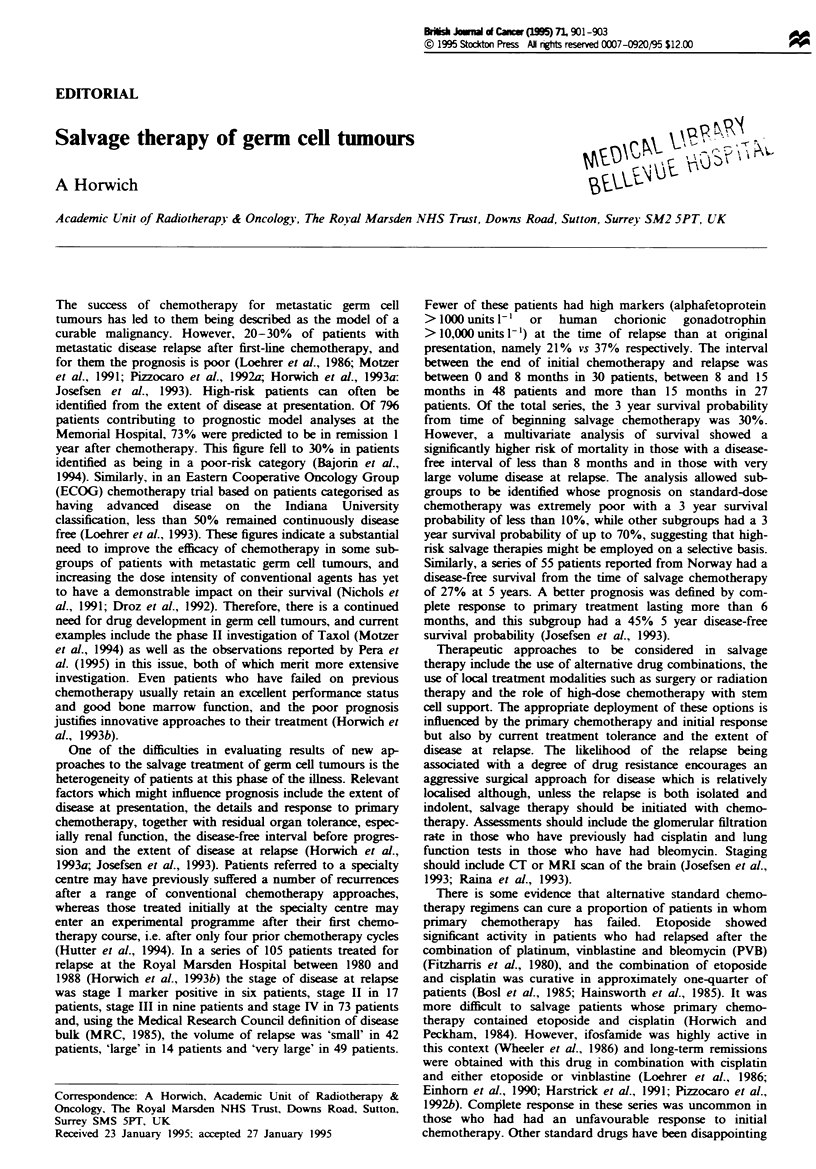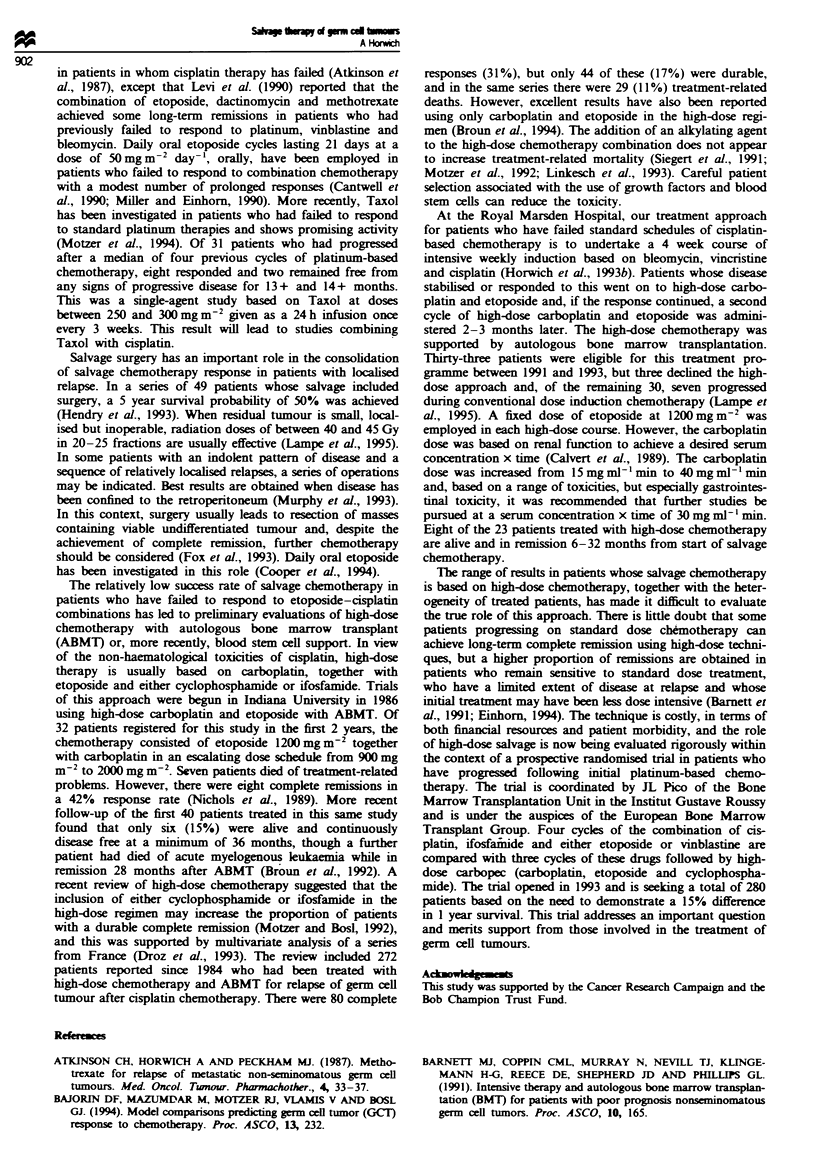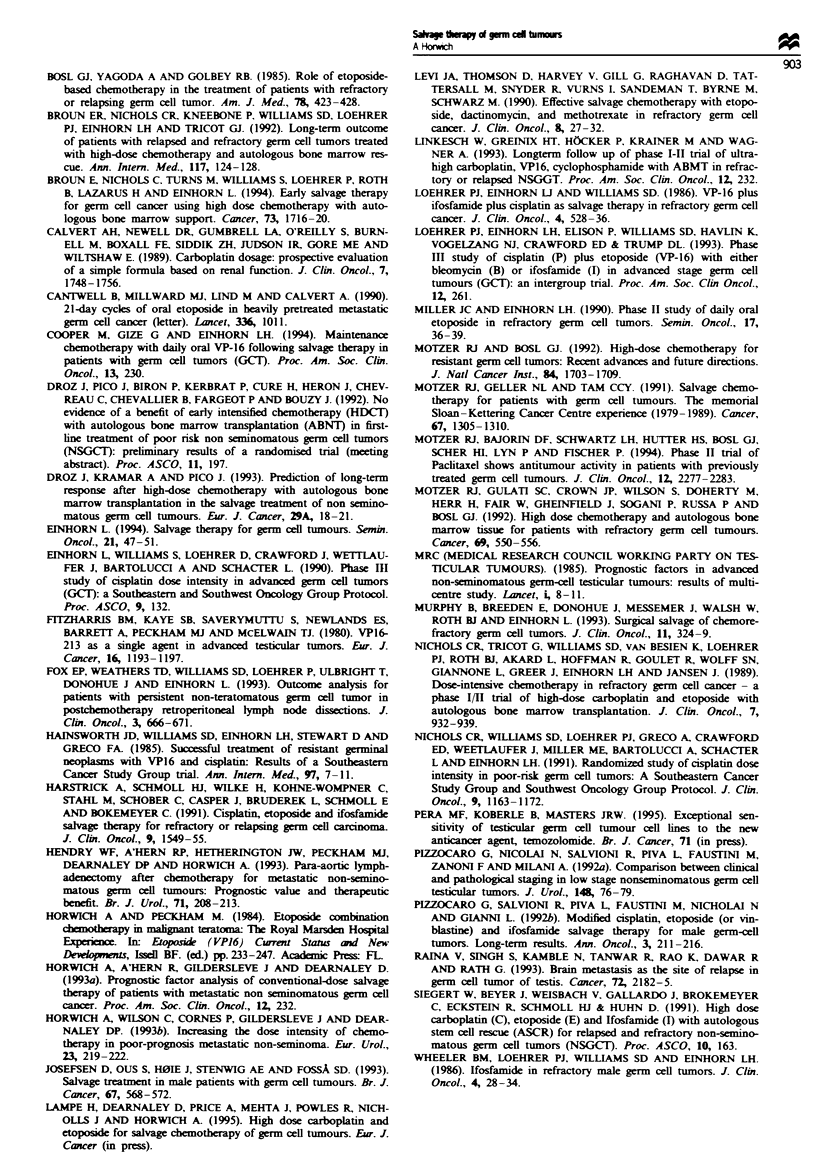# Salvage therapy of germ cell tumours.

**DOI:** 10.1038/bjc.1995.175

**Published:** 1995-05

**Authors:** A. Horwich


British Journal of Cancer (1995) 71, 901-903

© 1995 Stockton Press All rights reserved 0007-0920/95 $12.00

EDITORIAL

### Salvage therapy of germ cell tumours

MEDICAL LIBRARY
BELLEVUE HOSPITAL

A Horwich

*Academic Unit of Radiotherapy & Oncology, The Royal Marsden NHS Trust, Downs Road, Sutton, Surrey SM2 5PT, UK*

The success of chemotherapy for metastatic germ cell tumours has led to them being described as the model of a curable malignancy. However, 20–30% of patients with metastatic disease relapse after first-line chemotherapy, and for them the prognosis is poor (Loehrer *et al.*, 1986; Motzer *et al.*, 1991; Pizzocaro *et al.*, 1992*a*; Horwich *et al.*, 1993*a*: Josefsen *et al.*, 1993). High-risk patients can often be identified from the extent of disease at presentation. Of 796 patients contributing to prognostic model analyses at the Memorial Hospital, 73% were predicted to be in remission 1 year after chemotherapy. This figure fell to 30% in patients identified as being in a poor-risk category (Bajorin *et al.*, 1994). Similarly, in an Eastern Cooperative Oncology Group (ECOG) chemotherapy trial based on patients categorised as having advanced disease on the Indiana University classification, less than 50% remained continuously disease free (Loehrer *et al.*, 1993). These figures indicate a substantial need to improve the efficacy of chemotherapy in some subgroups of patients with metastatic germ cell tumours, and increasing the dose intensity of conventional agents has yet to have a demonstrable impact on their survival (Nichols *et al.*, 1991; Droz *et al.*, 1992). Therefore, there is a continued need for drug development in germ cell tumours, and current examples include the phase II investigation of Taxol (Motzer *et al.*, 1994) as well as the observations reported by Pera *et al.* (1995) in this issue, both of which merit more extensive investigation. Even patients who have failed on previous chemotherapy usually retain an excellent performance status and good bone marrow function, and the poor prognosis justifies innovative approaches to their treatment (Horwich *et al.*, 1993*b*).

One of the difficulties in evaluating results of new approaches to the salvage treatment of germ cell tumours is the heterogeneity of patients at this phase of the illness. Relevant factors which might influence prognosis include the extent of disease at presentation, the details and response to primary chemotherapy, together with residual organ tolerance, especially renal function, the disease-free interval before progression and the extent of disease at relapse (Horwich *et al.*, 1993*a*; Josefsen *et al.*, 1993). Patients referred to a specialty centre may have previously suffered a number of recurrences after a range of conventional chemotherapy approaches, whereas those treated initially at the specialty centre may enter an experimental programme after their first chemotherapy course, i.e. after only four prior chemotherapy cycles (Hutter *et al.*, 1994). In a series of 105 patients treated for relapse at the Royal Marsden Hospital between 1980 and 1988 (Horwich *et al.*, 1993*b*) the stage of disease at relapse was stage I marker positive in six patients, stage II in 17 patients, stage III in nine patients and stage IV in 73 patients and, using the Medical Research Council definition of disease bulk (MRC, 1985), the volume of relapse was 'small' in 42 patients, 'large' in 14 patients and 'very large' in 49 patients.

Correspondence: A Horwich, Academic Unit of Radiotherapy & Oncology, The Royal Marsden NHS Trust, Downs Road, Sutton, Surrey SMS 5PT, UK
Received 23 January 1995; accepted 27 January 1995

Fewer of these patients had high markers (alphafetoprotein $>1000$ units $l^{-1}$ or human chorionic gonadotrophin $>10,000$ units $l^{-1}$) at the time of relapse than at original presentation, namely 21% *vs* 37% respectively. The interval between the end of initial chemotherapy and relapse was between 0 and 8 months in 30 patients, between 8 and 15 months in 48 patients and more than 15 months in 27 patients. Of the total series, the 3 year survival probability from time of beginning salvage chemotherapy was 30%. However, a multivariate analysis of survival showed a significantly higher risk of mortality in those with a disease-free interval of less than 8 months and in those with very large volume disease at relapse. The analysis allowed subgroups to be identified whose prognosis on standard-dose chemotherapy was extremely poor with a 3 year survival probability of less than 10%, while other subgroups had a 3 year survival probability of up to 70%, suggesting that high-risk salvage therapies might be employed on a selective basis. Similarly, a series of 55 patients reported from Norway had a disease-free survival from the time of salvage chemotherapy of 27% at 5 years. A better prognosis was defined by complete response to primary treatment lasting more than 6 months, and this subgroup had a 45% 5 year disease-free survival probability (Josefsen *et al.*, 1993).

Therapeutic approaches to be considered in salvage therapy include the use of alternative drug combinations, the use of local treatment modalities such as surgery or radiation therapy and the role of high-dose chemotherapy with stem cell support. The appropriate deployment of these options is influenced by the primary chemotherapy and initial response but also by current treatment tolerance and the extent of disease at relapse. The likelihood of the relapse being associated with a degree of drug resistance encourages an aggressive surgical approach for disease which is relatively localised although, unless the relapse is both isolated and indolent, salvage therapy should be initiated with chemotherapy. Assessments should include the glomerular filtration rate in those who have previously had cisplatin and lung function tests in those who have had bleomycin. Staging should include CT or MRI scan of the brain (Josefsen *et al.*, 1993; Raina *et al.*, 1993).

There is some evidence that alternative standard chemotherapy regimens can cure a proportion of patients in whom primary chemotherapy has failed. Etoposide showed significant activity in patients who had relapsed after the combination of platinum, vinblastine and bleomycin (PVB) (Fitzharris *et al.*, 1980), and the combination of etoposide and cisplatin was curative in approximately one-quarter of patients (Bosl *et al.*, 1985; Hainsworth *et al.*, 1985). It was more difficult to salvage patients whose primary chemotherapy contained etoposide and cisplatin (Horwich and Peckham, 1984). However, ifosfamide was highly active in this context (Wheeler *et al.*, 1986) and long-term remissions were obtained with this drug in combination with cisplatin and either etoposide or vinblastine (Loehrer *et al.*, 1986; Einhorn *et al.*, 1990; Harstrick *et al.*, 1991; Pizzocaro *et al.*, 1992*b*). Complete response in these series was uncommon in those who had had an unfavourable response to initial chemotherapy. Other standard drugs have been disappointing in patients in whom cisplatin therapy has failed (Atkinson *et al.*, 1987), except that Levi *et al.* (1990) reported that the combination of etoposide, dactinomycin and methotrexate achieved some long-term remissions in patients who had previously failed to respond to platinum, vinblastine and bleomycin. Daily oral etoposide cycles lasting 21 days at a dose of 50 mg m$^{-2}$ day$^{-1}$, orally, have been employed in patients who failed to respond to combination chemotherapy with a modest number of prolonged responses (Cantwell *et al.*, 1990; Miller and Einhorn, 1990). More recently, Taxol has been investigated in patients who had failed to respond to standard platinum therapies and shows promising activity (Motzer *et al.*, 1994). Of 31 patients who had progressed after a median of four previous cycles of platinum-based chemotherapy, eight responded and two remained free from any signs of progressive disease for 13+ and 14+ months. This was a single-agent study based on Taxol at doses between 250 and 300 mg m$^{-2}$ given as a 24 h infusion once every 3 weeks. This result will lead to studies combining Taxol with cisplatin.

Salvage surgery has an important role in the consolidation of salvage chemotherapy response in patients with localised relapse. In a series of 49 patients whose salvage included surgery, a 5 year survival probability of 50% was achieved (Hendry *et al.*, 1993). When residual tumour is small, localised but inoperable, radiation doses of between 40 and 45 Gy in 20–25 fractions are usually effective (Lampe *et al.*, 1995). In some patients with an indolent pattern of disease and a sequence of relatively localised relapses, a series of operations may be indicated. Best results are obtained when disease has been confined to the retroperitoneum (Murphy *et al.*, 1993). In this context, surgery usually leads to resection of masses containing viable undifferentiated tumour and, despite the achievement of complete remission, further chemotherapy should be considered (Fox *et al.*, 1993). Daily oral etoposide has been investigated in this role (Cooper *et al.*, 1994).

The relatively low success rate of salvage chemotherapy in patients who have failed to respond to etoposide–cisplatin combinations has led to preliminary evaluations of high-dose chemotherapy with autologous bone marrow transplant (ABMT) or, more recently, blood stem cell support. In view of the non-haematological toxicities of cisplatin, high-dose therapy is usually based on carboplatin, together with etoposide and either cyclophosphamide or ifosfamide. Trials of this approach were begun in Indiana University in 1986 using high-dose carboplatin and etoposide with ABMT. Of 32 patients registered for this study in the first 2 years, the chemotherapy consisted of etoposide 1200 mg m$^{-2}$ together with carboplatin in an escalating dose schedule from 900 mg m$^{-2}$ to 2000 mg m$^{-2}$. Seven patients died of treatment-related problems. However, there were eight complete remissions in a 42% response rate (Nichols *et al.*, 1989). More recent follow-up of the first 40 patients treated in this same study found that only six (15%) were alive and continuously disease free at a minimum of 36 months, though a further patient had died of acute myelogenous leukaemia while in remission 28 months after ABMT (Broun *et al.*, 1992). A recent review of high-dose chemotherapy suggested that the inclusion of either cyclophosphamide or ifosfamide in the high-dose regimen may increase the proportion of patients with a durable complete remission (Motzer and Bosl, 1992), and this was supported by multivariate analysis of a series from France (Droz *et al.*, 1993). The review included 272 patients reported since 1984 who had been treated with high-dose chemotherapy and ABMT for relapse of germ cell tumour after cisplatin chemotherapy. There were 80 complete responses (31%), but only 44 of these (17%) were durable, and in the same series there were 29 (11%) treatment-related deaths. However, excellent results have also been reported using only carboplatin and etoposide in the high-dose regimen (Broun *et al.*, 1994). The addition of an alkylating agent to the high-dose chemotherapy combination does not appear to increase treatment-related mortality (Siegert *et al.*, 1991; Motzer *et al.*, 1992; Linkesch *et al.*, 1993). Careful patient selection associated with the use of growth factors and blood stem cells can reduce the toxicity.

At the Royal Marsden Hospital, our treatment approach for patients who have failed standard schedules of cisplatin-based chemotherapy is to undertake a 4 week course of intensive weekly induction based on bleomycin, vincristine and cisplatin (Horwich *et al.*, 1993*b*). Patients whose disease stabilised or responded to this went on to high-dose carboplatin and etoposide and, if the response continued, a second cycle of high-dose carboplatin and etoposide was administered 2–3 months later. The high-dose chemotherapy was supported by autologous bone marrow transplantation. Thirty-three patients were eligible for this treatment programme between 1991 and 1993, but three declined the high-dose approach and, of the remaining 30, seven progressed during conventional dose induction chemotherapy (Lampe *et al.*, 1995). A fixed dose of etoposide at 1200 mg m$^{-2}$ was employed in each high-dose course. However, the carboplatin dose was based on renal function to achieve a desired serum concentration × time (Calvert *et al.*, 1989). The carboplatin dose was increased from 15 mg ml$^{-1}$ min to 40 mg ml$^{-1}$ min and, based on a range of toxicities, but especially gastrointestinal toxicity, it was recommended that further studies be pursued at a serum concentration × time of 30 mg ml$^{-1}$ min. Eight of the 23 patients treated with high-dose chemotherapy are alive and in remission 6–32 months from start of salvage chemotherapy.

The range of results in patients whose salvage chemotherapy is based on high-dose chemotherapy, together with the heterogeneity of treated patients, has made it difficult to evaluate the true role of this approach. There is little doubt that some patients progressing on standard dose chemotherapy can achieve long-term complete remission using high-dose techniques, but a higher proportion of remissions are obtained in patients who remain sensitive to standard dose treatment, who have a limited extent of disease at relapse and whose initial treatment may have been less dose intensive (Barnett *et al.*, 1991; Einhorn, 1994). The technique is costly, in terms of both financial resources and patient morbidity, and the role of high-dose salvage is now being evaluated rigorously within the context of a prospective randomised trial in patients who have progressed following initial platinum-based chemotherapy. The trial is coordinated by JL Pico of the Bone Marrow Transplantation Unit in the Institut Gustave Roussy and is under the auspices of the European Bone Marrow Transplant Group. Four cycles of the combination of cisplatin, ifosfamide and either etoposide or vinblastine are compared with three cycles of these drugs followed by high-dose carbopec (carboplatin, etoposide and cyclophosphamide). The trial opened in 1993 and is seeking a total of 280 patients based on the need to demonstrate a 15% difference in 1 year survival. This trial addresses an important question and merits support from those involved in the treatment of germ cell tumours.

**Acknowledgements**
This study was supported by the Cancer Research Campaign and the Bob Champion Trust Fund.

**References**

ATKINSON CH, HORWICH A AND PECKHAM MJ. (1987). Methotrexate for relapse of metastatic non-seminomatous germ cell tumours. *Med. Oncol. Tumour. Pharmachother.*, **4**, 33–37.

BAJORIN DF, MAZUMDAR M, MOTZER RJ, VLAMIS V AND BOSL GJ. (1994). Model comparisons predicting germ cell tumor (GCT) response to chemotherapy. *Proc. ASCO*, **13**, 232.

BARNETT MJ, COPPIN CML, MURRAY N, NEVILL TJ, KLINGEMANN H-G, REECE DE, SHEPHERD JD AND PHILLIPS GL. (1991). Intensive therapy and autologous bone marrow transplantation (BMT) for patients with poor prognosis nonseminomatous germ cell tumors. *Proc. ASCO*, **10**, 165.

BOSL GJ, YAGODA A AND GOLBEY RB. (1985). Role of etoposide-based chemotherapy in the treatment of patients with refractory or relapsing germ cell tumor. *Am. J. Med.*, **78**, 423–428.

BROUN ER, NICHOLS CR, KNEEBONE P, WILLIAMS SD, LOEHRER PJ, EINHORN LH AND TRICOT GJ. (1992). Long-term outcome of patients with relapsed and refractory germ cell tumors treated with high-dose chemotherapy and autologous bone marrow rescue. *Ann. Intern. Med.*, **117**, 124–128.

BROUN E, NICHOLS C, TURNS M, WILLIAMS S, LOEHRER P, ROTH B, LAZARUS H AND EINHORN L. (1994). Early salvage therapy for germ cell cancer using high dose chemotherapy with autologous bone marrow support. *Cancer*, **73**, 1716–20.

CALVERT AH, NEWELL DR, GUMBRELL LA, O'REILLY S, BURNELL M, BOXALL FE, SIDDIK ZH, JUDSON IR, GORE ME AND WILTSHAW E. (1989). Carboplatin dosage: prospective evaluation of a simple formula based on renal function. *J. Clin. Oncol.*, **7**, 1748–1756.

CANTWELL B, MILLWARD MJ, LIND M AND CALVERT A. (1990). 21-day cycles of oral etoposide in heavily pretreated metastatic germ cell cancer (letter). *Lancet*, **336**, 1011.

COOPER M, GIZE G AND EINHORN LH. (1994). Maintenance chemotherapy with daily oral VP-16 following salvage therapy in patients with germ cell tumors (GCT). *Proc. Am. Soc. Clin. Oncol.*, **13**, 230.

DROZ J, PICO J, BIRON P, KERBRAT P, CURE H, HERON J, CHEVREAU C, CHEVALLIER B, FARGEOT P AND BOUZY J. (1992). No evidence of a benefit of early intensified chemotherapy (HDCT) with autologous bone marrow transplantation (ABNT) in first-line treatment of poor risk non seminomatous germ cell tumors (NSGCT): preliminary results of a randomised trial (meeting abstract). *Proc. ASCO*, **11**, 197.

DROZ J, KRAMAR A AND PICO J. (1993). Prediction of long-term response after high-dose chemotherapy with autologous bone marrow transplantation in the salvage treatment of non seminomatous germ cell tumours. *Eur. J. Cancer*, **29A**, 18–21.

EINHORN L. (1994). Salvage therapy for germ cell tumours. *Semin. Oncol.*, **21**, 47–51.

EINHORN L, WILLIAMS S, LOEHRER D, CRAWFORD J, WETTLAUFER J, BARTOLUCCI A AND SCHACTER L. (1990). Phase III study of cisplatin dose intensity in advanced germ cell tumors (GCT): a Southeastern and Southwest Oncology Group Protocol. *Proc. ASCO*, **9**, 132.

FITZHARRIS BM, KAYE SB, SAVERYMUTTU S, NEWLANDS ES, BARRETT A, PECKHAM MJ AND McELWAIN TJ. (1980). VP16-213 as a single agent in advanced testicular tumors. *Eur. J. Cancer*, **16**, 1193–1197.

FOX EP, WEATHERS TD, WILLIAMS SD, LOEHRER P, ULBRIGHT T, DONOHUE J AND EINHORN L. (1993). Outcome analysis for patients with persistent non-teratomatous germ cell tumor in postchemotherapy retroperitoneal lymph node dissections. *J. Clin. Oncol.*, **3**, 666–671.

HAINSWORTH JD, WILLIAMS SD, EINHORN LH, STEWART D AND GRECO FA. (1985). Successful treatment of resistant germinal neoplasms with VP16 and cisplatin: Results of a Southeastern Cancer Study Group trial. *Ann. Intern. Med.*, **97**, 7–11.

HARSTRICK A, SCHMOLL HJ, WILKE H, KOHNE-WOMPNER C, STAHL M, SCHOBER C, CASPER J, BRUDEREK L, SCHMOLL E AND BOKEMEYER C. (1991). Cisplatin, etoposide and ifosfamide salvage therapy for refractory or relapsing germ cell carcinoma. *J. Clin. Oncol.*, **9**, 1549–55.

HENDRY WF, A'HERN RP, HETHERINGTON JW, PECKHAM MJ, DEARNALEY DP AND HORWICH A. (1993). Para-aortic lymphadenectomy after chemotherapy for metastatic non-seminomatous germ cell tumours: Prognostic value and therapeutic benefit. *Br. J. Urol.*, **71**, 208–213.

HORWICH A AND PECKHAM M. (1984). Etoposide combination chemotherapy in malignant teratoma: The Royal Marsden Hospital Experience. In: *Etoposide (VP16) Current Status and New Developments*, Issell BF. (ed.) pp. 233–247. Academic Press: FL.

HORWICH A, A'HERN R, GILDERSLEVE J AND DEARNALEY D. (1993*a*). Prognostic factor analysis of conventional-dose salvage therapy of patients with metastatic non seminomatous germ cell cancer. *Proc. Am. Soc. Clin. Oncol.*, **12**, 232.

HORWICH A, WILSON C, CORNES P, GILDERSLEVE J AND DEARNALEY DP. (1993*b*). Increasing the dose intensity of chemotherapy in poor-prognosis metastatic non-seminoma. *Eur. Urol.*, **23**, 219–222.

JOSEFSEN D, OUS S, HØIE J, STENWIG AE AND FOSSÅ SD. (1993). Salvage treatment in male patients with germ cell tumours. *Br. J. Cancer*, **67**, 568–572.

LAMPE H, DEARNALEY D, PRICE A, MEHTA J, POWLES R, NICHOLLS J AND HORWICH A. (1995). High dose carboplatin and etoposide for salvage chemotherapy of germ cell tumours. *Eur. J. Cancer* (in press).

LEVI JA, THOMSON D, HARVEY V, GILL G, RAGHAVAN D, TATTERSALL M, SNYDER R, VURNS I, SANDEMAN T, BYRNE M, SCHWARZ M. (1990). Effective salvage chemotherapy with etoposide, dactinomycin, and methotrexate in refractory germ cell cancer. *J. Clin. Oncol.*, **8**, 27–32.

LINKESCH W, GREINIX HT, HÖCKER P, KRAINER M AND WAGNER A. (1993). Longterm follow up of phase I-II trial of ultra-high carboplatin, VP16, cyclophosphamide with ABMT in refractory or relapsed NSGGT. *Proc. Am. Soc. Clin. Oncol.*, **12**, 232.

LOEHRER PJ, EINHORN LJ AND WILLIAMS SD. (1986). VP-16 plus ifosfamide plus cisplatin as salvage therapy in refractory germ cell cancer. *J. Clin. Oncol.*, **4**, 528–36.

LOEHRER PJ, EINHORN LH, ELISON P, WILLIAMS SD, HAVLIN K, VOGELZANG NJ, CRAWFORD ED & TRUMP DL. (1993). Phase III study of cisplatin (P) plus etoposide (VP-16) with either bleomycin (B) or ifosfamide (I) in advanced stage germ cell tumours (GCT): an intergroup trial. *Proc. Am. Soc. Clin Oncol.*, **12**, 261.

MILLER JC AND EINHORN LH. (1990). Phase II study of daily oral etoposide in refractory germ cell tumors. *Semin. Oncol.*, **17**, 36–39.

MOTZER RJ AND BOSL GJ. (1992). High-dose chemotherapy for resistant germ cell tumors: Recent advances and future directions. *J. Natl Cancer Inst.*, **84**, 1703–1709.

MOTZER RJ, GELLER NL AND TAM CCY. (1991). Salvage chemotherapy for patients with germ cell tumours. The memorial Sloan–Kettering Cancer Centre experience (1979–1989). *Cancer*, **67**, 1305–1310.

MOTZER RJ, BAJORIN DF, SCHWARTZ LH, HUTTER HS, BOSL GJ, SCHER HI, LYN P AND FISCHER P. (1994). Phase II trial of Paclitaxel shows antitumour activity in patients with previously treated germ cell tumours. *J. Clin. Oncol.*, **12**, 2277–2283.

MOTZER RJ, GULATI SC, CROWN JP, WILSON S, DOHERTY M, HERR H, FAIR W, GHEINFIELD J, SOGANI P, RUSSA P AND BOSL GJ. (1992). High dose chemotherapy and autologous bone marrow tissue for patients with refractory germ cell tumours. *Cancer*, **69**, 550–556.

MRC (MEDICAL RESEARCH COUNCIL WORKING PARTY ON TESTICULAR TUMOURS). (1985). Prognostic factors in advanced non-seminomatous germ-cell testicular tumours: results of multicentre study. *Lancet*, **i**, 8–11.

MURPHY B, BREEDEN E, DONOHUE J, MESSEMER J, WALSH W, ROTH BJ AND EINHORN L. (1993). Surgical salvage of chemorefractory germ cell tumors. *J. Clin. Oncol.*, **11**, 324–9.

NICHOLS CR, TRICOT G, WILLIAMS SD, VAN BESIEN K, LOEHRER PJ, ROTH BJ, AKARD L, HOFFMAN R, GOULET R, WOLFF SN, GIANNONE L, GREER J, EINHORN LH AND JANSEN J. (1989). Dose-intensive chemotherapy in refractory germ cell cancer – a phase I/II trial of high-dose carboplatin and etoposide with autologous bone marrow transplantation. *J. Clin. Oncol.*, **7**, 932–939.

NICHOLS CR, WILLIAMS SD, LOEHRER PJ, GRECO A, CRAWFORD ED, WEETLAUFER J, MILLER ME, BARTOLUCCI A, SCHACTER L AND EINHORN LH. (1991). Randomized study of cisplatin dose intensity in poor-risk germ cell tumors: A Southeastern Cancer Study Group and Southwest Oncology Group Protocol. *J. Clin. Oncol.*, **9**, 1163–1172.

PERA MF, KOBERLE B, MASTERS JRW. (1995). Exceptional sensitivity of testicular germ cell tumour cell lines to the new anticancer agent, temozolomide. *Br. J. Cancer*, **71** (in press).

PIZZOCARO G, NICOLAI N, SALVIONI R, PIVA L, FAUSTINI M, ZANONI F AND MILANI A. (1992*a*). Comparison between clinical and pathological staging in low stage nonseminomatous germ cell testicular tumors. *J. Urol.*, **148**, 76–79.

PIZZOCARO G, SALVIONI R, PIVA L, FAUSTINI M, NICHOLAI N AND GIANNI L. (1992*b*). Modified cisplatin, etoposide (or vinblastine) and ifosfamide salvage therapy for male germ-cell tumors. Long-term results. *Ann. Oncol.*, **3**, 211–216.

RAINA V, SINGH S, KAMBLE N, TANWAR R, RAO K, DAWAR R AND RATH G. (1993). Brain metastasis as the site of relapse in germ cell tumor of testis. *Cancer*, **72**, 2182–5.

SIEGERT W, BEYER J, WEISBACH V, GALLARDO J, BROKEMEYER C, ECKSTEIN R, SCHMOLL HJ & HUHN D. (1991). High dose carboplatin (C), etoposide (E) and Ifosfamide (I) with autologous stem cell rescue (ASCR) for relapsed and refractory non-seminomatous germ cell tumors (NSGCT). *Proc. ASCO*, **10**, 163.

WHEELER BM, LOEHRER PJ, WILLIAMS SD AND EINHORN LH. (1986). Ifosfamide in refractory male germ cell tumors. *J. Clin. Oncol.*, **4**, 28–34.